# Radiomics to predict the mortality of patients with rheumatoid arthritis-associated interstitial lung disease: A proof-of-concept study

**DOI:** 10.3389/fmed.2022.1069486

**Published:** 2023-01-09

**Authors:** Vincenzo Venerito, Andreina Manfredi, Giuseppe Lopalco, Marlea Lavista, Giulia Cassone, Arnaldo Scardapane, Marco Sebastiani, Florenzo Iannone

**Affiliations:** ^1^Rheumatology Unit, Department of Emergency and Organ Transplantation, University of Bari Aldo Moro, Bari, Italy; ^2^Rheumatology Unit, Azienda Ospedaliera Policlinico di Modena, University of Modena and Reggio Emilia, Modena, Italy; ^3^Radiology Unit, Department of Interdisciplinary Medicine, University of Bari Aldo Moro, Bari, Italy

**Keywords:** radiomics, rheumatoid arthritis-associated interstitial lung disease, high-resolution computed tomography, biomarker, LASSO

## Abstract

**Objectives:**

Patients with rheumatoid arthritis (RA) and interstitial lung disease (ILD) have increased mortality compared to the general population and factors capable of predicting RA-ILD long-term clinical outcomes are lacking. In oncology, radiomics allows the quantification of tumour phenotype by analysing the characteristics of medical images. Using specific software, it is possible to segment organs on high-resolution computed tomography (HRCT) images and extract many features that may uncover disease characteristics that are not detected by the naked eye. We aimed to investigate whether features from whole lung radiomic analysis of HRCT may alone predict mortality in RA-ILD patients.

**Methods:**

High-resolution computed tomographies of RA patients from January 2012 to March 2022 were analyzed. The time between the first available HRCT and the last follow-up visit or ILD-related death was recorded. We performed a volumetric analysis in 3D Slicer, automatically segmenting the whole lungs and trachea *via* the Lung CT Analyzer. A LASSO-Cox model was carried out by considering ILD-related death as the outcome variable and extracting radiomic features as exposure variables.

**Results:**

We retrieved the HRCTs of 30 RA-ILD patients. The median survival time (interquartile range) was 48 months (36–120 months). Thirteen out of 30 (43.33%) patients died during the observation period. Whole line segmentation was fast and reliable. The model included either the median grey level intensity within the whole lung segmentation [high-resolution (HR) 9.35, 95% CI 1.56–55.86] as a positive predictor of death and the 10th percentile of the number of included voxels (HR 0.20, 95% CI 0.05–0.84), the voxel-based pre-processing information (HR 0.23, 95% CI 0.06–0.82) and the flatness (HR 0.42, 95% CI 0.18–0.98), negatively correlating to mortality. The correlation of grey level values to their respective voxels (HR 1.52 95% CI 0.82–2.83) was also retained as a confounder.

**Conclusion:**

Radiomic analysis may predict RA-ILD patients’ mortality and may promote HRCT as a digital biomarker regardless of the clinical characteristics of the disease.

## Introduction

Patients with rheumatoid arthritis (RA) have decreased survival compared to the general population (1, 2). RA-associated interstitial lung disease (RA-ILD) is a common extra-articular manifestation of RA. The median survival of RA-ILD patients is about 3–7 years, which is markedly reduced compared to RA patients without ILD and the general population (3–5). Detection of RA-ILD varies widely by different research methods. Consequently, the reported prevalence of RA-ILD reflects such high variance, with studies based on chest computed tomography (CT) scans indicating RA-ILD presence in 10–30% of patients. Despite the recent advance with potential diagnostic biomarkers such as the MUC5B promotor variant (6) or sound analysis of vesicular murmur (7), there is a scarcity of factors capable of predicting RA-ILD long-term clinical outcomes. In oncology, radiomics allows for comprehensive tumour phenotype quantification by examining medical images’ characteristics. In brief, using specific software, it is possible to segment organs on CT images to extract a massive number of features that have the potential to uncover disease characteristics that fail to be seen by the naked eye. The hypothesis of radiomics is that the distinctive imaging features between disease forms may help make a prognosis and predict the therapeutic response for various conditions, thus providing valuable information for personalised therapy and patient management (8). We aimed to investigate whether features from whole lung radiomic analysis of high-resolution computed tomography (HRCT) might alone predict mortality in RA-ILD patients.

## Materials and methods

We retrieved the consecutive HRCTs of patients affected with RA according to 2010 EULAR/ACR criteria (9), and RA-ILD followed at the Rheumatology departments of two Italian tertiary centres from January 2012 to March 2022. To be included in such retrospective analysis, HRCTs had to have been carried out at one of the Radiology departments of the same centres, and the DICOM files had to have been stored in the respective picture archiving and communication systems (PACS). We recorded the time interval between the first available HRCT and the last follow-up visit or eventual death for physician-reported ILD-related causes on death certificates for each patient. We also recorded clinical and demographic characteristics together with rheumatoid factor (RF) and anti-cyclic citrullinated peptide antibodies (ACPA) status. We excluded patients with known overlapping connective tissue disease or secondary Sjogren’s syndrome. In particular, we considered only HRCT examinations with 0.625–1.25-mm slice thickness and full-inspiration scans from the lung apices to below the costo-phrenic angles. Both centres used the following parameters: tube voltage set at 120 kV; tube current: fixed mAs depending on the patient body weight; pitch: 0.8 or more, adjusted on the seriousness of patient dyspnoea, collimation: 0.6 mm; images were reconstructed from raw data using a slice thickness of 0.6 mm with an index of 0.4 mm using an edge-enhancing algorithm. All images were viewed at a window setting optimised for assessment of the lung parenchyma (width 1,500 HU; level −700 HU).

The study was approved and reviewed by the local Ethical Committee (Biopure registry, IRB Approval n.5940, Azienda Ospedaliera Universitaria di Bari). All patients gave their written informed consent.

### Segmentation and radiomic features extraction

We performed a volumetric analysis and visualisation in 3D Slicer^1^, automatically segmenting the whole lungs and trachea *via* the Lung CT Analyzer project^2^. As previously described elsewhere (10), such an extension allows fully reproducible automated whole lungs and trachea segmentation using 13 manually marked points. Three points were placed in axial and coronal views inside the right and the left lung, and one point in the trachea (further details in Supplementary material). For each HRCT, segmentation was repeated twice by a rheumatologist and a radiologist in order to check for radiomic feature stability (see below). Two expert radiologists then reviewed the latter segmentations to confirm that all lung parenchyma had been appropriately isolated.

The PyRadiomics platform was developed for cancer research with the US National Cancer Institute grant 5U24CA194354 (8). It can extract radiomic data from medical imaging loading, pre-process the image and segmentation maps, calculate features using the different feature classes, and return results as continuous variables in CSV format. In a Python 3.9 environment, the PyRadiomics API (version 3.01) was implemented to extract 120 features for segmented regions (see Supplementary material for further technical details). Such features, including skewness and kurtosis, together with more specific texture-related ones, comply with feature definitions as described by the imaging biomarker standardization initiative (IBSI) (8).

Collinearity was checked, and all redundant features were removed (i.e., with a correlation higher than 0.8 as an absolute value, see Supplementary material). We carried out feature z-score standardisation before the next step.

### Statistics

Intraclass correlation coefficient (ICC) was used to test radiomic feature stability by comparing the abovementioned segmentations. The survival function was plotted with the Kaplan–Meier estimate together with the at-risk table. Cox survival analysis and least absolute shrinkage and selection operator (LASSO) regression univariable analyses were performed to assess the association of each radiomic feature with death. Values of *p* < 0.20 were selected as candidate variables. The method of LASSO was used to determine predictors as such procedures demonstrated superior accuracy than stepwise elimination (11). As a proof-of-concept study, demographics and disease characteristics were not included in the regression model to demonstrate the feasibility and investigate the association of features alone with RA patients’ death. Cox-Snell residuals were plotted against the Nelson–Aalen cumulative hazard rate function to test the model’s reliability. Stata 17 (StataCorp, TX, USA), together with Python 3.9 herein invoked with Pystata API, numpy 1.22, pandas 1.4.3, and scikit-learn 1.1.2 libraries, were used on a terminal powered by an Apple™ Silicon M1Max with 64 GB RAM.

## Results

**FIGURE 1:**
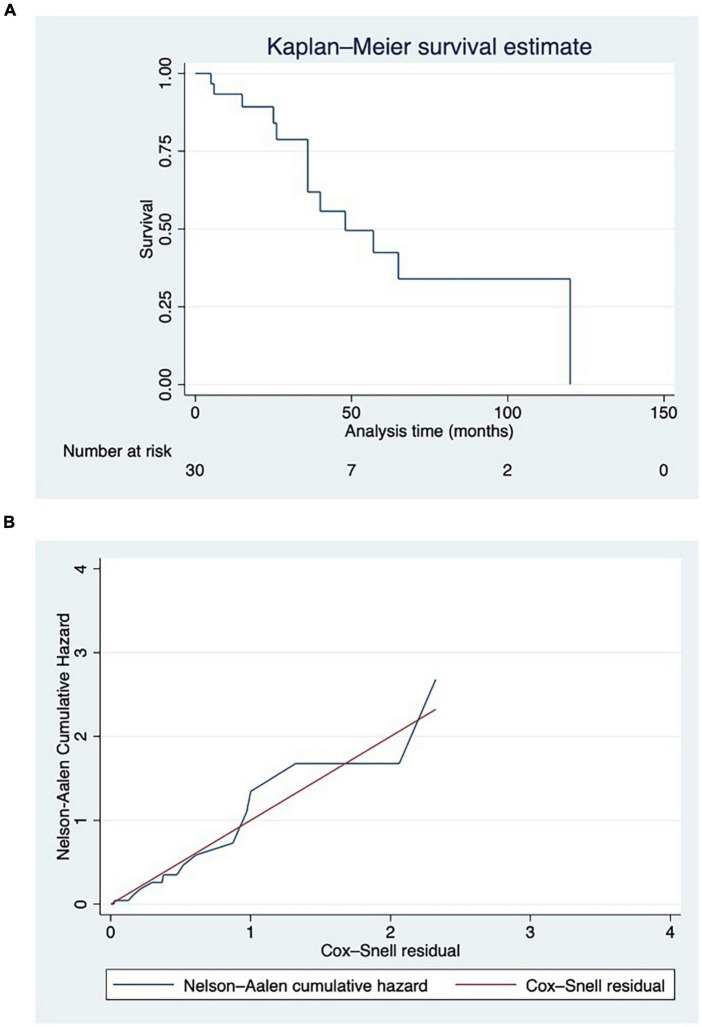
**(A)** Survival function displayed using Kaplan–Meier estimate, with at-risk table. Deaths have been reported in parentheses. **(B)** Cox-Snell residuals plotted against the Nelson–Aalen cumulative hazard rate function to test the reliability of the model. Hazard function followed the 45-degree line very closely except for huge values of time.

We retrieved HRCTs of 30 RA-ILD patients, 11 males (36.67%) with median age [interquartile range (IQR)] of 72 (65–78) years at the instrumental examination. They had established RA with median disease duration (IQR) of 132 (65–278) months. ACPA positivity was found in 25 out of 30 patients (83.33%), whereas RF-positive individuals were found in 18 out of 29 (62.97%). Usual interstitial pneumonia (UIP) was the most frequent finding (18/30, 60%) at HRCT, followed by unclassifiable patterns (8/30, 26, 67%). Non-specific interstitial pneumonia (NSIP) in two patients (6.67%), whereas organising pneumonia (OP) and lymphocytic interstitial pneumonia (LIP) were found in one patient, respectively (3.33%, for both). Thirteen out of 30 patients (43.33%) were on methotrexate at HRCT examination, whereas 19 out of them (63.33%) were treated with biologic agents and two with baricitinib (6.67%). Complete patient characteristics and ILD patterns are shown in Table 1. The mean follow-up time (± standard deviation) was 37.99 ± 29.50 months median survival time (MST—IQR) was 48 months (36–120 months). Thirteen out of 30 (43.33%) patients died during the observation period, and the cause of death was attributed to ILD as judged by the physician. Kaplan–Meier survival function was plotted in Figure 1A.

**TABLE 1:** Patient characteristics.

	Available observations	
Male, *n* (%)	30	11 (36.67)
Age at HRCT, years, median (IQR)	30	72 (65–78)
RA disease duration, month, median (IQR)	30	132 (65–278)
RF positivity, *n* (%)	29	18 (62.07)
ACPA positivity *n* (%)	30	25 (83.33)
ILD pattern at HRCT, (*n*%)	30	
UIP		18 (60)
NSIP		2 (2.67)
LIP		1 (3.33)
OP		1 (3.33)
Unclassifiable		8 (26.67)
Therapy at HRCT, *n* (%)	30	
MTX		17 (56.67)
Tocilizumab		7 (23.33)
Abatacept		6 (0.20)
Rituximab		5 (16.66)
Anakinra		1 (3.33)
Baricitinib		2 (6.67)

ACPA, anti-cyclic citrullinated peptide antibodies; HRCT, high-resolution computed tomography; LIP, lymphocytic interstitial pneumonia; ILD, interstitial lung disease; IQR, interquartile range; MTX, methotrexate; NSIP, non-specific interstitial pneumonia; OP, organising pneumonia; RA, rheumatoid arthritis; RF, rheumatoid factor; UIP, usual interstitial pneumonia.

The death cause was acute ILD exacerbations in 38.46% of cases (5/13) and pneumonia in 23.07% (3/13). Finally, another 38.46% (5/13) of death certificates and health records reported generic “RA-ILD” as the cause of death.

**FIGURE 2:**
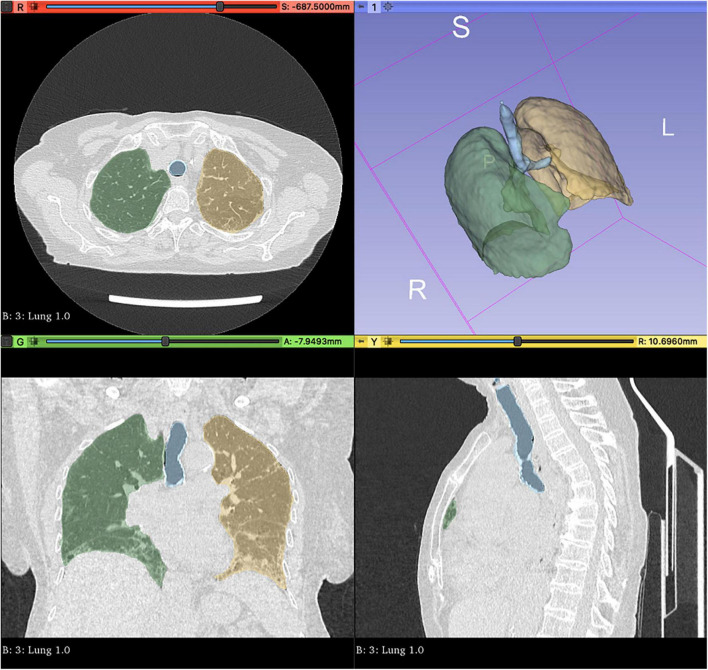
Automated whole lungs and trachea segmentation using 3D Slicer (http://www.slicer.org) *via* the Lung CT Analyzer project (https://github.com/rbumm/SlicerLungCTAnalyzer/).

The automatic segmentation allowed for the comprehensive and precise isolation of RA-ILD lung parenchyma and upper airways coherent with radiologist judgement in all cases at the first attempt (Figure 2). The segmentation procedure took a mean 3.13 ± 2.11 min on average. Such a procedure showed excellent feature stability with ICC = 1, indicating perfect reliability between operators. After checking for collinearity (Supplementary Figure 4), only 22 features were retained (see Supplementary material for the full list).

After the LASSO-Cox procedure, the model included the features:

• The median grey level intensity within the whole lung segmentation mask [coded as original_firstorder_median, high-resolution (HR) 9.35, 95% CI 1.56–55.86].
• The 10th percentile of the number of voxels included in the whole lung segmentation mask (coded as original_firstorder_10Percentile HR 0.20, 95% CI 0.05–0.84).
• 3D Slicer voxel-based pre-processing information (coded as diagnostics_Imageoriginal_Mean, HR 0.23, 95% CI 0.06–0.82).
• Flatness—the relationship between the largest and smallest principal components in the whole lung mask (coded as original_shape_Flatness, HR 0.42, 95% CI 0.18–0.98).
• Correlation of grey level values to their respective voxels in the Grey level co-occurrence matrix (coded as original_glcm_Correlation, HR 1.52 95% CI 0.82–2.83), retained as a confounder.

We observed that the hazard function followed the 45-degree line very closely except for huge values of time (Figure 1B).

## Discussion

Patients with RA-ILD have an increased disease burden, reduced quality of life and physical function, severe respiratory symptoms, and worse RA disease. ILD also leads to substantial healthcare costs and interactions; 72% have an all-cause inpatient admission, and 76% have an all-cause emergency department visit. Finally, as mentioned above, they also experience excess mortality compared to the general population and patients with RA without ILD (1, 2, 12). In this scenario, it is conceivable that improving patient management and follow-up is of utmost importance. Many efforts have been made to improve early ILD diagnosis. Investigating the role of potential perturbations to pulmonary mucosa in airways seems to be an approach with concrete potential. In fact, the MUC5B promotor variant in RA patients has been associated with a threefold increased risk for ILD (6).

Furthermore, an algorithm (Vector) to detect the presence of velcro crackles in pulmonary sounds showed promising results for screening RA patients suspected of ILD and who should be directed to HRCT for the diagnosis (12). But, only a few tools seem to have the potential for driving treatment strategy (13–15), mostly involving the shifts in levels of selected serum proteins, such as CXCL11/I-TAC and matrix metalloproteinase-13, which may not be available in routine clinical practice. Radiomics has been helpful in phenotyping cancers (8, 16, 17). It can quantify a large panel of phenotypic characteristics, such as shape and texture, potentially reflecting biologic properties like intra- and inter-tumour heterogeneities and related distinct treatment responses (8). An advantage of radiomics is the standardised procedure for extraction, relying on open-source libraries but dependent upon the segmentation of the region of interest of medical images, which can be obtained by an expert radiologist or machine learning algorithms.

In this study, we applied radiomics to baseline HRCT of the lungs of RA patients with ILD to search for predictors of mortality. To this end, we used an automated segmentation tool allowing whole lung and trachea segmentation. In literature, such a procedure appeared precise and accounted for high user reliability (10, 18). The same was true when we used this tool on ILD parenchyma in all our patients, despite two different CT machines and slice thickness, enabling us to extract 120 radiomic features for our analysis systematically. We found a model including n.5 radiomic features associated with RA_ILD patients’ death, with good fitting except for large values of the time. Adjusting for original_glcm_Correlation, the radiomic feature original_firstorder_median was positively correlated, whereas original_firstorder_10Percentile, diagnostics_Imageoriginal_Mean and original_shape_Flatness radiomic features were negatively correlated to ILD-related mortality.

Our results confirm that lung imaging has prognostic potential. In this regard, they are somewhat consistent with the report from Oh et al. using quantitative HRCT (QCT) scores to predict mortality in RA-ILD (19). They analyzed a retrospective cohort of 144 RA-ILD patients diagnosed at a single centre between 1999 and 2015. All patients had HRCT performed at RA-ILD diagnosis, and baseline clinical data included autoimmunity status, inflammatory markers, pulmonary function tests, and medication use. To assess the baseline, HRCTs were assessed by an automated quantification system (AQS) that divided each lung image into small regions of interest and scored each region of interest for the presence of reticulation, architectural distortion, ground-glass opacification, and honeycombing. By combining these scores, the AQS generated a quantitative lung fibrosis score (QLF) capable of predicting RA-ILD outcomes but also independently associated with mortality after adjustment for age, baseline erythrocyte sedimentation rate (ESR), and pulmonary function tests (20). Although similar in its fundamentals, the radiomics approach offers several advantages. Radiomic features may be used with genomic data leading to the so-called radiogenomics, which showed potential for both diagnosis and prognosis in oncology research (8). This appears particularly interesting in RA-ILD given the recent findings about MUC5B- related ILD risk (6). Furthermore, the whole lung radiomics does not miss information from apparent healthy parenchyma, contributing to the image and tissue characteristics analysis that a naked-eye approach would never consider. Finally, *de novo* radiomic feature extraction may be easily standardised and does not rely on algorithm training.

We must acknowledge some weaknesses of our study. First, as the proof-of-concept method, we analyzed a small-sized retrospective cohort. As already mentioned, we did not apply non-linear machine learning methods, which might provide better modelling of radiomic features than linear methods. This preliminary study demonstrated that several radiomics features are predictors of RA-ILD patients’ mortality in the absence of demographics and disease-related characteristics. It is also conceivable that using a large, annotated dataset of radiomics features from HRCT-segmented whole-lung parenchyma, it could be possible to discriminate RA-ILD from interstitial lung involvement of different connective tissue diseases. More extensive studies with internal and external validation on independent cohorts are needed to confirm that a radiomics approach could be adopted in routine clinical practice. Further research is required to enable HRCT to provide digital biomarkers of RA-ILD outcomes.

## Data availability statement

The raw data supporting the conclusions of this article will be made available by the authors, without undue reservation.

## Ethics statement

The studies involving human participants were reviewed and approved by the Comitato Etico Interregionale. The patients/participants provided their written informed consent to participate in this study.

## Author contributions

VV, AM, and MS designed the study and wrote the manuscript. VV and AS did the data analysis. ML and GC gathered the patient data. FI supervised each step of the whole study. All authors contributed to the article and approved the submitted version.

## Footnotes

1
http://www.slicer.org

2
https://github.com/rbumm/SlicerLungCTAnalyzer/
