# Supplementary material for: Radiomics to predict the mortality of patients with rheumatoid arthritis-associated interstitial lung disease: A proof-of-concept study
Source: Front Med (Lausanne). 2023 Jan 9;9:1069486. doi: 10.3389/fmed.2022.1069486 (PMC9870287; doi:10.3389/fmed.2022.1069486)
Supplement: Supplementary file 1 [file Data_Sheet_1.docx]

**SUPPLEMENTARY MATERIAL**

**Segmentation procedure**

LungCTAnalyzer extension (2) (<https://github.com/rbumm/SlicerLungCTAnalyzer>) was used after importing the CT datasets in 3DSlicer software. The lung masks were automatically segmented by the “LungCTSegmenter” extension using 13 manually marked points. Three points were placed in axial and coronal views inside the right and the left lung and one point in the trachea (Supplementary Figures 1 and 2). The same procedures were repeated twice (specifically by VV, MD Rheumatologist and AS, MD Radiologist). All lung masks were verified visually independently by a third radiologist (MC) (Supplementary Figure 3), no corrections were needed.


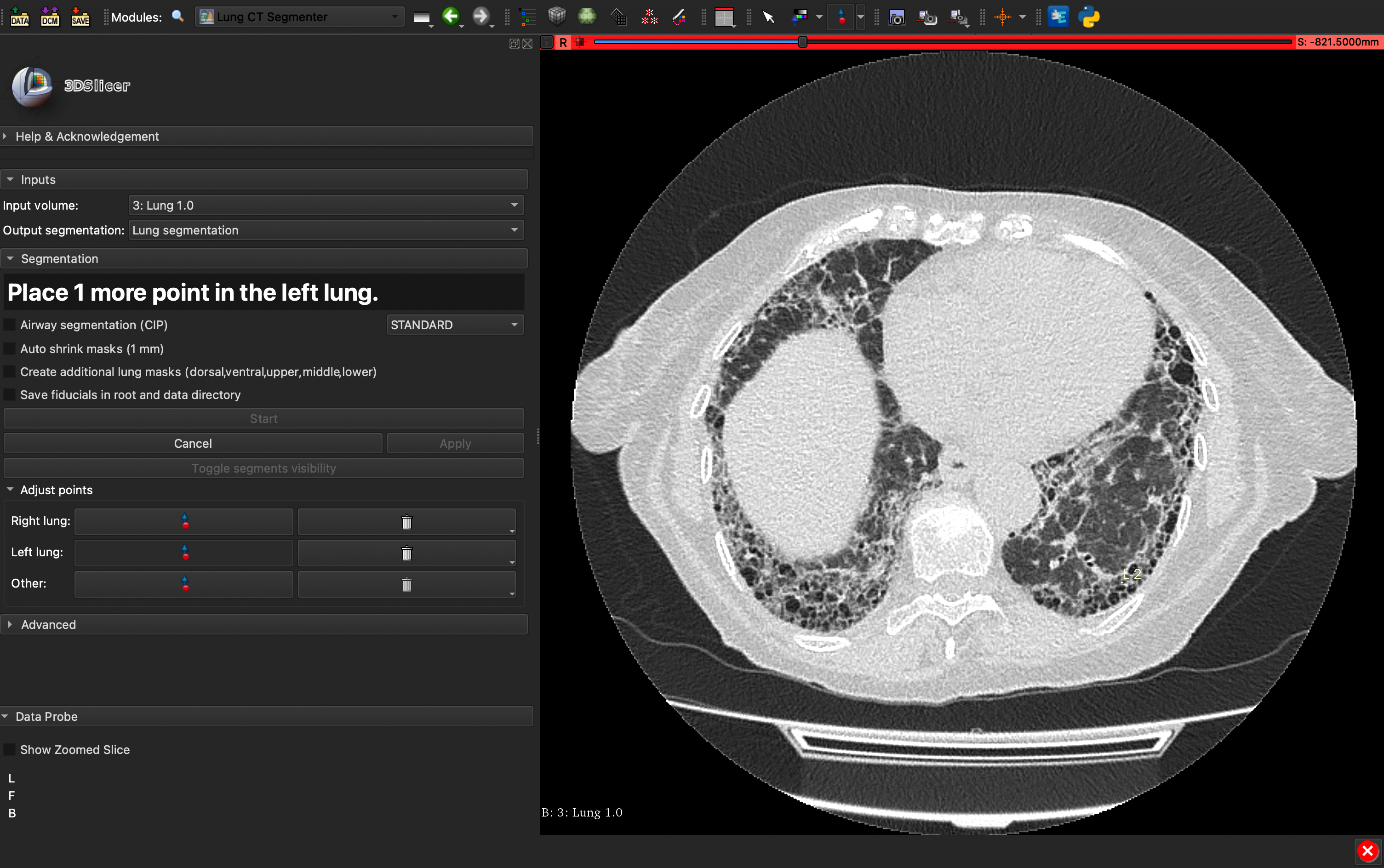


Supplementary Figure 1. Automated segmentation procedure in coronal view


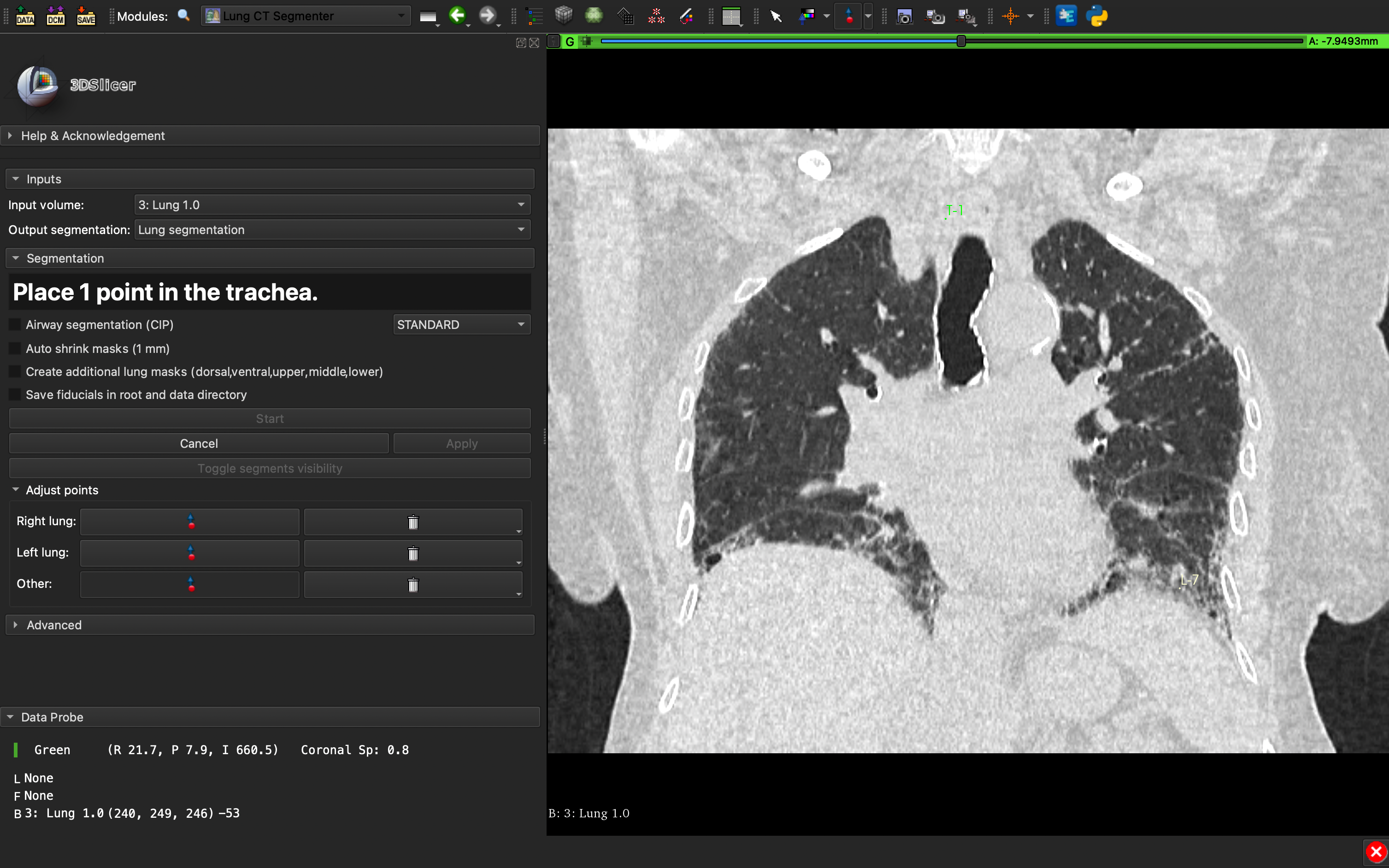


Supplementary Figure 2. Automated segmentation procedure in axial view,


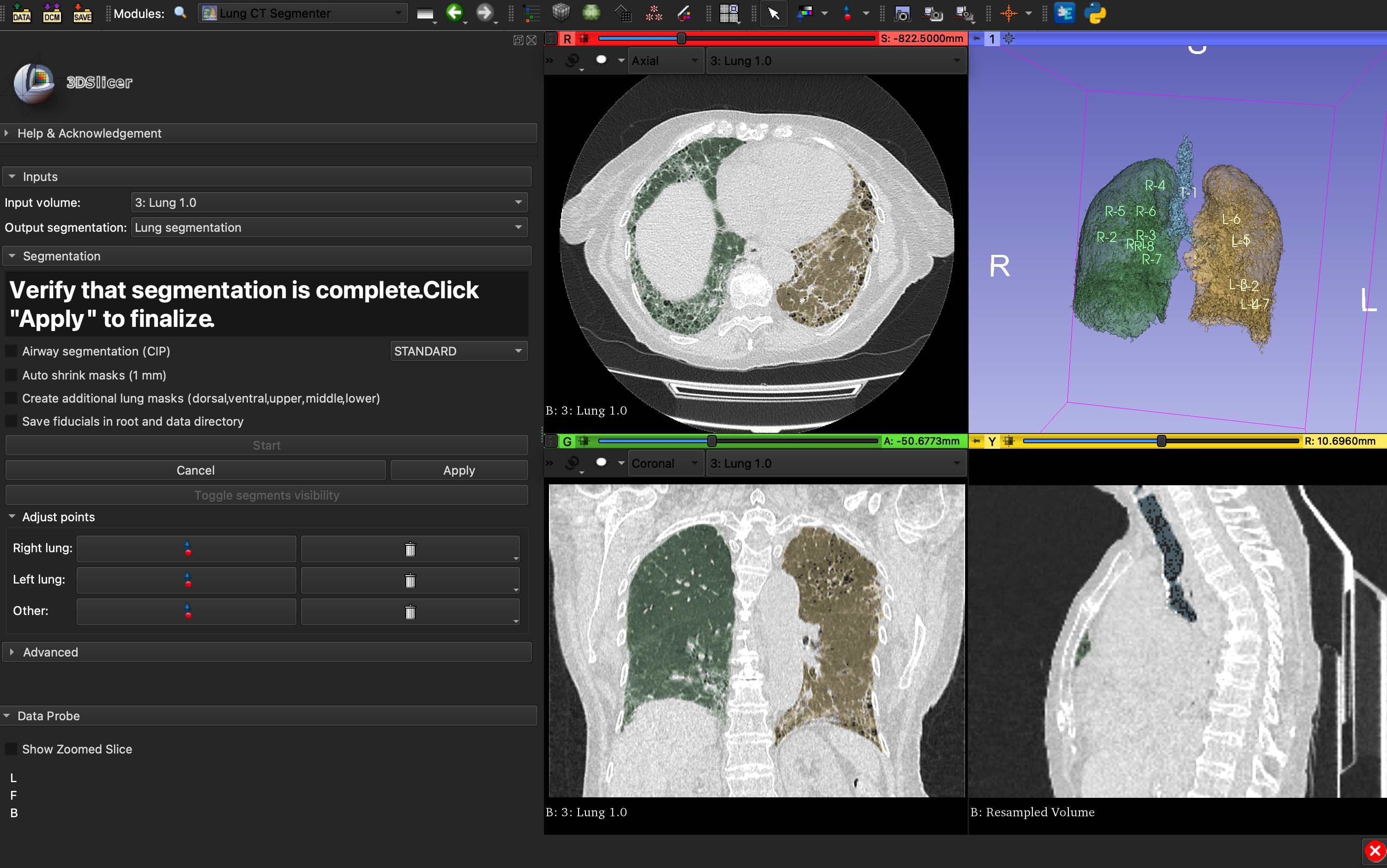


Supplementary Figure 3. Automated segmentation providing precise lung and trachea masks.

**Radiomic Features Extraction**

We deployed Pyradiomics API v.3.01 as per documentation in a Jupyter notebook with Python 3.9 Kernel as previously reported (1,2). Both lung images and masks were in NRRD format.

All extracted featured are available in the documentation of PyRadiomics API v.3.01 (1). Such features are in compliance with feature definitions as described by the Imaging Biomarker Standardization Initiative (IBSI) (3).

Here we provide rapid hyperlinks reporting each extracted feature and its definition.

- [**First Order Statistics**](https://pyradiomics.readthedocs.io/en/v3.0.1/features.html#radiomics.firstorder.RadiomicsFirstOrder) (19 features)
- [**Shape-based (3D)**](https://pyradiomics.readthedocs.io/en/v3.0.1/features.html#radiomics.shape.RadiomicsShape) (16 features)
- [**Shape-based (2D)**](https://pyradiomics.readthedocs.io/en/v3.0.1/features.html#radiomics.shape2D.RadiomicsShape2D) (10 features)
- [**Gray Level Cooccurence Matrix**](https://pyradiomics.readthedocs.io/en/v3.0.1/features.html#radiomics.glcm.RadiomicsGLCM) (24 features)
- [**Gray Level Run Length Matrix**](https://pyradiomics.readthedocs.io/en/v3.0.1/features.html#radiomics.glrlm.RadiomicsGLRLM) (16 features)
- [**Gray Level Size Zone Matrix**](https://pyradiomics.readthedocs.io/en/v3.0.1/features.html#radiomics.glszm.RadiomicsGLSZM) (16 features)
- [**Neighbouring Gray Tone Difference Matrix**](https://pyradiomics.readthedocs.io/en/v3.0.1/features.html#radiomics.ngtdm.RadiomicsNGTDM) (5 features)
- [**Gray Level Dependence Matrix**](https://pyradiomics.readthedocs.io/en/v3.0.1/features.html#radiomics.gldm.RadiomicsGLDM) (14 features)

~~
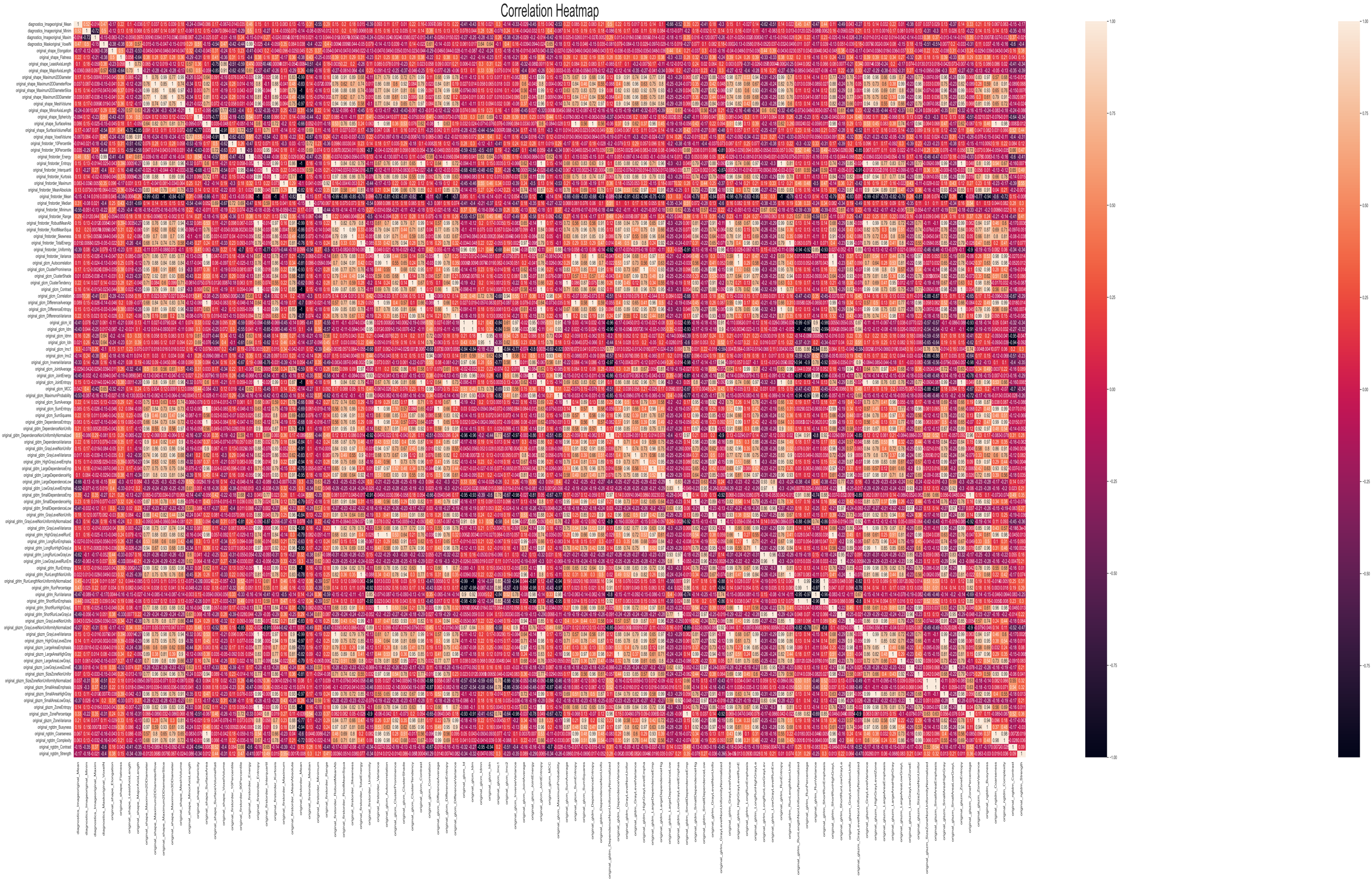
~~Supplementary Figure 4. Autocorrelation Matrix.

Final feature dataset after collinearity check:

- 'diagnostics_Imageoriginal_Mean',
- 'diagnostics_Imageoriginal_Minim',
- 'diagnostics_Imageoriginal_Maxim',
- 'diagnostics_Maskoriginal_VoxelN',
- 'original_shape_Elongation',
- 'original_shape_Flatness',
- 'original_shape_LeastAxisLength',
- 'original_shape_MajorAxisLength',
- 'original_shape_Maximum2DDiameter',
- 'original_shape_Maximum2DDiameterRow',
- 'original_shape_MinorAxisLength',
- 'original_shape_Sphericity',
- 'original_firstorder_10Percentile',
- 'original_firstorder_90Percentile',
- 'original_firstorder_Maximum',
- 'original_firstorder_Median',
- 'original_firstorder_Minimum',
- 'original_firstorder_Uniformity',
- 'original_glcm_Correlation',
- 'original_glcm_Idmn', '
- original_gldm_LargeDependenceLow',
- 'original_ngtdm_Strength'

**REFERENCES**

1. van Griethuysen JJM, Fedorov A, Parmar C, Hosny A, Aucoin N, Narayan V, Beets-Tan RGH, Fillion-Robin J-C, Pieper S, Aerts HJWL. Computational Radiomics System to Decode the Radiographic Phenotype. *Cancer Res* (2017) 77:e104–e107. doi: 10.1158/0008-5472.CAN-17-0339

2. Risoli C, Nicolò M, Colombi D, Moia M, Rapacioli F, Anselmi P, Michieletti E, Ambrosini R, Di Terlizzi M, Grazioli L, et al. Different Lung Parenchyma Quantification Using Dissimilar Segmentation Software: A Multi-Center Study for COVID-19 Patients. *Diagnostics (Basel)* (2022) 12:1501. doi: 10.3390/diagnostics12061501

3. Zwanenburg A, Leger S, Vallières M, Löck S. Image biomarker standardisation initiative. *Radiology* (2020) 295:328–338. doi: 10.1148/radiol.2020191145
